# Smoothness metric during reach-to-grasp after stroke: part 2. longitudinal association with motor impairment

**DOI:** 10.1186/s12984-021-00937-w

**Published:** 2021-09-24

**Authors:** Mique Saes, Mohamed Irfan Mohamed Refai, Joost van Kordelaar, Bouke L. Scheltinga, Bert-Jan F. van Beijnum, Johannes B. J. Bussmann, Jaap H. Buurke, Peter H. Veltink, Carel G. M. Meskers, Erwin E. H. van Wegen, Gert Kwakkel

**Affiliations:** 1 Department of Rehabilitation Medicine, Amsterdam UMC, Vrije Universiteit Amsterdam, Amsterdam Movement Sciences, Amsterdam Neuroscience, de Boelelaan 1117, Location VUmc, PO Box 7057, 1007 MB Amsterdam, The Netherlands; 2 Department of Biomedical Signals & Systems, Technical Medical Centre, University of Twente, Enschede, The Netherlands; 3 Department of Rehabilitation Medicine, Erasmus MC, University Medical Centre Rotterdam, Rotterdam, The Netherlands; 4 Department of Physical Therapy and Human Movement Sciences, Feinberg School of Medicine, Northwestern University, Chicago, Il USA; 5 Rehabilitation Technology, Roessingh Research and Development, Enschede, The Netherlands; 6 Department of Neurorehabilitation, Amsterdam Rehabilitation Research Centre, Reade, Amsterdam, The Netherlands

**Keywords:** Stroke, Longitudinal study, Kinematics, Upper extremity, Recovery, Smoothness

## Abstract

**Background:**

The cause of smoothness deficits as a proxy for quality of movement post stroke is currently unclear. Previous simulation analyses showed that spectral arc length (SPARC) is a valid metric for investigating smoothness during a multi-joint goal-directed reaching task. The goal of this observational study was to investigate how SPARC values change over time, and whether SPARC is longitudinally associated with the recovery from motor impairments reflected by the Fugl-Meyer motor assessment of the upper extremity (FM-UE) in the first 6 months after stroke.

**Methods:**

Forty patients who suffered a first-ever unilateral ischemic stroke (22 males, aged 58.6 ± 12.5 years) with upper extremity paresis underwent kinematic and clinical measurements in weeks 1, 2, 3, 4, 5, 8, 12, and 26 post stroke. Clinical measures included amongst others FM-UE. SPARC was obtained by three-dimensional kinematic measurements using an electromagnetic motion tracking system during a reach-to-grasp movement. Kinematic assessments of 12 healthy, age-matched individuals served as reference. Longitudinal linear mixed model analyses were performed to determine SPARC change over time, compare smoothness in patients with reference values of healthy individuals, and establish the longitudinal association between SPARC and FM-UE scores.

**Results:**

SPARC showed a significant positive longitudinal association with FM-UE (B: 31.73, 95%-CI: [27.27 36.20], *P* < 0.001), which encompassed significant within- and between-subject effects (B: 30.85, 95%-CI: [26.28 35.41], *P* < 0.001 and B: 50.59, 95%-CI: [29.97 71.21], *P* < 0.001, respectively). Until 5 weeks post stroke, progress of time contributed significantly to the increase in SPARC and FM-UE scores (*P* < 0.05), whereafter they levelled off. At group level, smoothness was lower in patients who suffered a stroke compared to healthy subjects at all time points (*P* < 0.05).

**Conclusions:**

The present findings show that, after stroke, recovery of smoothness in a multi-joint reaching task and recovery from motor impairments are longitudinally associated and follow a similar time course. This suggests that the reduction of smoothness deficits quantified by SPARC is a proper objective reflection of recovery from motor impairment, as reflected by FM-UE, probably driven by a common underlying process of spontaneous neurological recovery early post stroke.

**Supplementary Information:**

The online version contains supplementary material available at 10.1186/s12984-021-00937-w.

## Introduction

Motor impairments of the upper extremity are estimated to occur in about 80% of patients who survived a stroke [1, 2]. These impairments are characterized by weakness, diminished dexterity, spatial and temporal discontinuity (i.e., lack of smoothness), and abnormal stereotypic patterns of muscle activation or muscle synergies during goal-directed movements [3, 4]. Spontaneous motor recovery occurs mainly in the first 10 weeks post stroke, depending on stroke severity [5]. Smoothness of movements, for example during reaching, is an important indicator of quality of movement (QoM) [6–9], which is valuable for computational models of neurological recovery when studying motor control after stroke [10–12]. Enhanced smoothness has been argued to reflect improved sensorimotor coordination and movement proficiency [13, 14]. Unfortunately, the underlying neurophysiological mechanisms of post-stroke smoothness deficits during multi-joint movements such as reaching are poorly understood [11].

Several theories have been proposed, for example less smooth movements may reflect unstable co-contractions between agonists and antagonists due to a lack of reciprocal inhibition [15, 16]. In line with this, muscle activity patterns observed during reaching after stroke were shown to be more synchronized [17, 18]. An EMG study suggested that a reduced motor unit discharge rate post stroke would explain the decreased smoothness [19]. Buma et al. found an association between an increase in jerk and additional cortical recruitment in secondary sensorimotor areas as shown by fMRI in subjects with subacute stroke, which supports the hypothesis of enhanced online feedback corrections to prevent movement errors during upper limb reaching early after stroke [20]. One may also hypothesize that the lack of smoothness is a reflection of increased segmentation of multi-joint movements [16], observed together with abnormal muscle synergies [21–23]. Although the underlying neurophysiological cause of smoothness deficits are unknown, improvement of smoothness deficits after stroke has been assumed to reflect neurological recovery. Therefore, one may hypothesize that recovery of smoothness will occur in the same time window as that of spontaneous neurological recovery post stroke. As a consequence, smoothness may serve as a fine-grained marker for measuring recovery of motor control early post stroke [10].

In our previous study, we showed that out of 32 different smoothness metrics which have been used in stroke studies, only *spectral arc length* (SPARC) [10] is a valid metric to reflect smoothness during a multi-joint reach-to-grasp movement [24]. The frequency spectrum of a movement is dependent on the sub-movements dispersed in time. Smooth movements are assumed to be composed of mainly low-frequency components, whereas less smooth movements show a larger amount of higher-frequency components and thereby show a more complex magnitude spectrum. The smoothness metric SPARC is based on the complexity of the shape of a Fourier magnitude spectrum of the velocity profile during a reaching task [25]. However, recovery of SPARC during reaching movements has not been investigated longitudinally early after stroke, nor its within-subject association with motor recovery measured with the Fugl-Meyer motor assessment of the upper extremity (FM-UE).

Assuming that recovery of smoothness reflects a decreasing segmentation of motor performance due to progressive blending of sub-movements [16], we hypothesized that an increase in SPARC values would be associated with recovery from motor impairments as measured with FM-UE. In addition, we hypothesized that SPARC would improve mainly in the early phase, whereafter it would level-off, within the time window of spontaneous neurological recovery. Therefore, the present paper addresses three key questions. First, whether smoothness, reflected by SPARC during a reach-to-grasp movement, is longitudinally associated with FM-UE scores in the first six months post stroke. Second, whether the observed time window of recovery of smoothness is in line with the time window of FM-UE recovery. Third, whether patients attain healthy reference values of smoothness within the first six months post stroke.

## Materials and methods

### Participants and procedures

Patients admitted to one of the acute stroke units of eleven participating hospitals in the Netherlands were screened. This prospective longitudinal multicentre cohort study, which was part of a translational research programme to explain plasticity after stroke (EXPLICIT-stroke [26]) included 40 patients who suffered a stroke (22 males, 18 females). Inclusion criteria were: (1) ≤ 1 week after a first-ever ischemic hemispheric stroke, as revealed by computerized axial tomography or magnetic resonance imaging scan; (2) being able to sit independently without trunk support for at least 30 s; (3) upper limb motor deficits, but with the ability to grasp objects within 3 weeks post stroke; (4) aged between 18 and 80 years; and (5) having provided written informed consent. Exclusion criteria were: (1) severe cognitive deficits (Mini-Mental State Examination score < 23); (2) comorbidities such as cardiac, pulmonary, orthopaedic or other neurological disorders; and (3) participating in other studies. By using their paretic arm, patients performed clinical assessments, as well as a 3-dimensional kinematic reach-to-grasp task to estimate their movement smoothness. This was done weekly between week 1 and week 5 post stroke, and at weeks 8, 12, and 26. Patients were allowed to receive movement therapy during the study.

Twelve age- and gender-matched healthy individuals without reported history of neurological and/or orthopaedic disorders (7 males, 5 females) were included to obtain healthy reference values for smoothness.

The EXPLICIT-stroke study was approved by the Medical Ethics Committee of the VU University medical centre, Amsterdam, The Netherlands, and carried out in accordance with the Code of Ethics of the World Medical Association, Declaration of Helsinki [27].

### Clinical assessment

A clinical measure of motor impairment commonly used in stroke studies is the Fugl-Meyer motor assessment of the upper extremity (FM-UE, range [0–66]), which shows excellent inter-rater and intra-rater reliability and construct validity [28]. Although the FM-UE originates from the evolution of abnormal muscle synergies [3, 29], it is also influenced by other impairments such as upper limb paresis [30], and is widely used to describe neurological motor impairment after stroke. Bamford classification [31] was used to establish the type of stroke; the National Institutes of Health Stroke Scale (NIHSS) [32] was used to assess the global neurological deficit; clinical assessments to determine functional ability included the Action Research Arm Test (ARAT) [33] of the paretic upper extremity and the Barthel Index (BI) [34]; sensory deficits were monitored by performing the Erasmus MC modification of the Nottingham Sensory Assessment (EmNSA) [35] of the upper extremity.

### Kinematic measurement

**Fig. 1:**
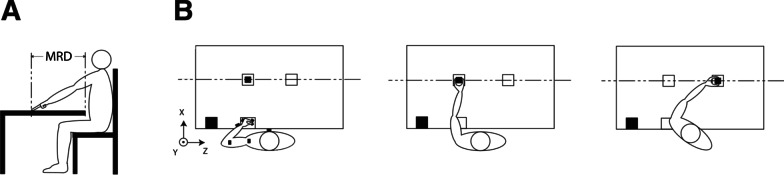
Kinematic measurement set-up. (A) Determination of the maximum reaching distance (MRD), also indicated by a dashed line. (B) Visualization of the task performance. Left panel: initial position and visualization of sensor placement on the subject. Middle panel: reaching forward towards the block (small black square), grasping the block between thumb and index finger. Right panel: lifting and moving the block without sliding and placing it at the indicated end position. The large black square at the corner of the table indicates the position of the electromagnetic source of the Polhemus Liberty system

Participants were seated on a chair, with their paretic hand placed in front of their shoulder on the edge of a table with a height of 76 cm. A wooden block of 5×5×5 cm (150 g) was placed in front of their shoulder at a participant-specific maximum reaching distance, obtained using the less affected arm. Participants were asked to reach towards the block, grasp the block with their thumb and index finger, lift it, and place it on the indicated position at the less affected body side (Fig. 1). Participants were instructed not to slide the block or upper limb over the table, but to move the hand through the air. Participants started after the experimenter gave a verbal “go” signal. During this movement, participants were not allowed to slide or twist over the seat of the chair, but were allowed to move their trunk away from the back of the chair if this was more comfortable. Each measurement involved recording seven repetitions. Healthy individuals performed the reach-to-grasp movement with their non-dominant hand.

Kinematic data were recorded using a portable electromagnetic motion tracking device (Polhemus Liberty) consisting of an electromagnetic source and seven motion sensors of size 2.3 × 2.8 × 1.5 cm. The source was placed on the edge of the table at the paretic side [22] (Fig. 1). Sensors were attached to the thorax, and to six segments of the paretic upper extremity (scapula, upper arm, forearm, hand, thumb, and index finger), using double-sided adhesive tape (Fig. 1). Only the data of sensors placed on the forearm and hand were used for the present study. The sampling frequency during the motion recordings was 240 Hz. All 3-dimensional kinematic assessments were conducted by one researcher (JvK). This portable setup enabled measurements at the participants’ place of residence (e.g. stroke unit in a hospital, rehabilitation centre, nursing home or their home situation), limiting the burden on patients in this longitudinal study.

### Kinematic data analysis

The reach-to-grasp part of the movement performed was extracted and analysed. The start of the movement was defined as the moment at which the hand sensor exceeded 5% of the maximum tangential speed during the forward reach [36]. The end of the reaching movement was defined as the moment at which the forearm sensor exceeded 5% of the maximum tangential speed for the first time during the displacement of the block [37]. Time series for displacement of the hand were filtered using a 2nd order recursive Butterworth low-pass filter with a cut-off frequency of 20 Hz. All computations were performed in MATLAB (2015b, The Mathworks, Natick, MA, USA). A detailed description of the computation of SPARC can be found in the first part of this twin paper [10, 24]. Higher SPARC values (i.e. less negative) reflect smoother movements.

### Statistical analysis

The longitudinal association between smoothness metric SPARC and FM-UE within the first six months post stroke, and their change over time, were both analysed using a linear mixed model.

For the first analysis, the smoothness metric SPARC served as independent variable, while FM-UE served as dependent variable. A random intercept was added for each individual to account for dependency within subjects. The regression coefficient of a regular longitudinal association is a combination of a within- and between-subject effect. These two effects can be distinguished by applying a hybrid model [38]. The between-subject covariate was determined as the individual average value of the smoothness metric over time, while the within-subject covariate was calculated as the observed value minus the individual average. The hybrid model results in two regression coefficients. The within-subject regression coefficient is the most interesting for the present analysis. It reflects whether the change of the dependent variable within a subject over time is associated with a change of the independent variable within a subject over time.

For the second analysis, the factor ‘week of measurement’ was included as the main fixed effect; a random intercept per individual was added to account for dependency within subjects. Two separate models were applied for SPARC and FM-UE as dependent variables.

SPARC values of patients who suffered a stroke were compared with reference values obtained from healthy participants at every time point using independent samples t-tests. Multiple testing was accounted for using the Holm-Bonferroni method [39].

Statistical analyses were performed using IBM SPSS Statistic for Windows, version 26.0 (IBM Corp., Armonk, NY, USA). For each regression model, the distribution of residuals was tested for normality by inspecting histograms and Q-Q plots.

## Results

### Participants

Table 1 displays the baseline characteristics of the 40 included patients who suffered a stroke (22 males; mean age ± SD: 58.6 ± 12.5 years) and the 12 healthy age- and gender-matched participants (7 males; mean age ± SD: 52.8 ± 5.9 years). All recruited patients had the ability to grasp the object in the third week post stroke. Twenty patients were able to perform the kinematic assessment starting in the first week after stroke onset, 13 starting in the second week, and seven starting in the third week post stroke.

**Table 1:** Participant characteristics at baseline

Characteristics	Values^a^
Stroke patients (N = 40)	
Age (years)	58.6 ± 12.5
Sex (male/female)	22/18
Most affected body side (left/right)	25/15
Hand dominance (left/right/forced right)	2/37/1
Bamford classification (LACI/PACI/TACI)	29/9/2
Time post stroke of the first clinical assessment (days)	7.3 ± 2.9
Clinical scores at baseline (week 1 post stroke)	
FM-UE (0–66)	43.5 (29.3–54.5)
FM-UE_arm_ (0–52)	34 (21.0–44.0)
NIHSS (42–0)	4.0 (2.0–5.0)
ARAT (0–57)	25.0 (7.3–36.0)
BI (0–20)	15.0 (11.0–17.0)
EmNSA (0–40)	40.0 (34.8–40.0)
Healthy participants (N = 12)	
Age (years)	52.8 ± 5.9
Sex (male/female)	7/5

N, number of participants; LACI, lacunar anterior circular infarct; PACI, partial anterior circular infarct; TACI, total anterior circular infarct; FM-UE, Fugl-Meyer motor assessment of the upper extremity; FM-UE_arm_, FM-UE without hand function scores; NIHSS, National Institutes of Health Stroke Scale; ARAT, Action Research Arm Test; BI, Barthel Index; EmNSA, Erasmus MC modified Nottingham Sensory Assessment of the upper extremity

^a^Values are number, mean ± standard deviation or median (interquartile range)

### Longitudinal association between SPARC and FM-UE

**Fig. 2:**
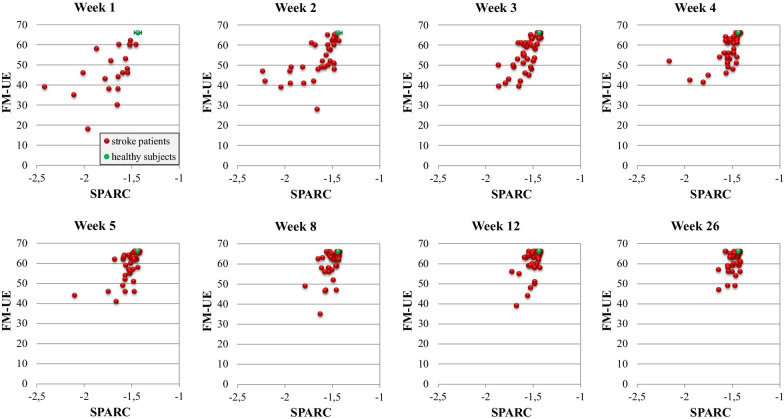
Smoothness against motor performance at each measurement moment post stroke. Scatter plots of Spectral Arc Length (SPARC) against Fugl-Meyer motor assessment score of the Upper Extremity (FM-UE) at each measurement moment. Red solid dots represent data of stroke patients. Green dots with error bars represent the average value and standard deviation of the healthy age- and gender-matched controls

**Fig. 3:**
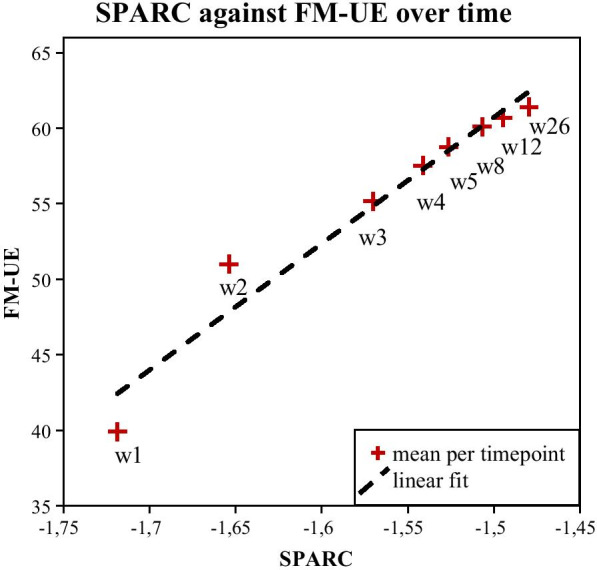
Smoothness against motor performance change in parallel post stroke. Mean Spectral Arc Length (SPARC) against mean Fugl-Meyer motor assessment score of the Upper Extremity (FM-UE) at each measurement moment indicated by red crosses. The dashed black line concerns a linear fit

SPARC showed a significant positive longitudinal association with FM-UE (B: 31.73, 95%-CI: [27.27 36.20], *P* < 0.001). The hybrid model showed that this association encompassed a significant within- and between-subject effect (B: 30.85, 95%-CI: [26.28 35.41], *P* < 0.001 and B: 50.59, 95%-CI: [29.97 71.21], *P* < 0.001, respectively). Figure 2 shows smoothness against motor impairment at each measurement moment. Figure 3 shows for each measurement moment the average smoothness in the investigated population against the average motor impairment score. These figures visualize that when patients show recovery of smoothness, they also show recovery from motor impairment in parallel. Moreover, the kinematic metric SPARC suffers less from a ceiling effect compared to the clinical measure FM-UE.

### Change over time and comparison with reference values

**Fig. 4:**
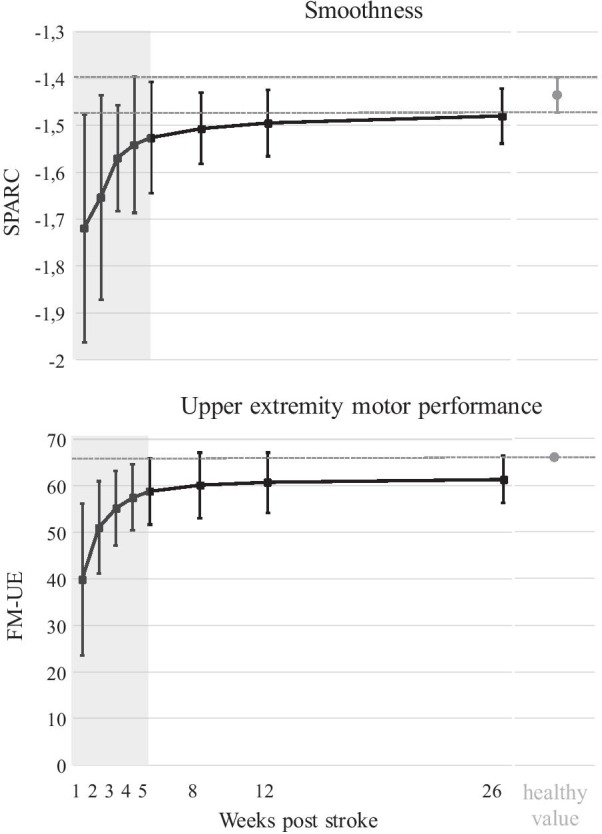
Smoothness and motor performance of the upper extremity as a function of time post stroke. The black squares and corresponding error bars represent the average and standard deviation of SPARC and FM-UE. Shaded area indicates the time period for which the contribution of time was significant (i.e., until week 5 post stroke) for SPARC and FM-UE. Thereafter, recovery levelled off. The grey dots and broken lines display the average and standard deviation of reference values of age- and gender-matched healthy individuals, from which patients who suffered a stroke deviated at all time points. Abbreviations: SPARC, spectral arc length; FM-UE, Fugl-Meyer motor assessment of the upper extremity

Figure 4 shows the change of SPARC and FM-UE over time post stroke, and how the values of patients who suffered a stroke compare with the reference values of the healthy individuals. The effect of time after stroke was significant for weeks 1 to 4 after stroke for SPARC and FM-UE (*P* < 0.05, Table 2). SPARC showed a gradual increase over time towards the reference values of the healthy individuals; it levelled off in week 5 (Table 2), yet remained lower in the patients who suffered a stroke than the age-matched healthy individuals (*P* < 0.05/N_s_, Table 3). FM-UE showed an increase over time and levelled off in week 5 (Table 2).

**Table 2:** Regression coefficients of SPARC and FM-UE relative to week 26 post stroke

Time points	SPARC			FM-UE		
	B	95%-CI	P	B	95%-CI	P
(Intercept)	− 1.48	[− 1.52 − 1.43]	**< 0.001**	61.40	[58.60 64.20]	** < 0.001**
Week 1	− 0.26	[− 0.20 − 0.32]	** < 0.001**	− 21.48	[− 18.76 − 24.19]	** < 0.001**
Week 2	− 0.18	[− 0.13 − 0.23]	** < 0.001**	− 9.89	[− 7.11 − 12.67]	** < 0.001**
Week 3	− 0.09	[− 0.04 − 0.14]	** < 0.001**	− 5.46	[− 2.47 − 8.46]	** < 0.001**
Week 4	− 0.06	[− 0.01 − 0.11]	**0.025**	− 3.25	[− 0.32 − 6.18]	**0.030**
Week 5	− 0.05	[0.00 − 0.10]	0.062	− 2.62	[0.12 − 5.35]	0.061
Week 8	− 0.03	[0.02 − 0.08]	0.290	− 0.94	[1.91 − 3.79]	0.515
Week 12	− 0.01	[0.04 − 0.06]	0.558	− 0.66	[2.12 − 3.43]	0.643
Week 26	0ª	–	**–**	0ª	–	**–**

B, regression coefficient; 95%-CI, 95% confidence interval; P, probability value; SPARC, spectral arc length; FM-UE, Fugl-Meyer motor assessment of the upper extremity

ªThis parameter is set to 0 because it is redundant. Significant values are indicated in bold font. A P-value below 0.05 indicates a significant difference from the reference time point (week 26). For both SPARC and FM-UE, the contribution of time was significant until week 5 post stroke

**Table 3:** Reach-to-grasp smoothness of stroke patients compared to healthy reference values

	SPARC			
	Mean	SD	t(df)	*P*
Week 1	− 1.719	0.243	3.98(30)	**< 0.001**
Week 2	− 1.654	0.218	3.42(43)	**0.001**
Week 3	− 1.570	0.114	3.98(50)	** < 0.001**
Week 4	− 1.541	0.145	2.47(50)	**0.017**
Week 5	− 1.526	0.118	2.59(50)	**0.013**
Week 8	− 1.507	0.076	3.11(50)	**0.003**
Week 12	− 1.495	0.070	2.77(50)	**0.008**
Week 26	− 1.480	0.059	2.43(50)	**0.019**
Healthy age- and gender-matched individuals	− 1.436	0.038	–	–

SPARC, spectral arc length (less negative values reflect smoother movements); SD, standard deviation; t, t-statistic of the independent samples t-test; df, degrees of freedom; P, probability value. Significant probability values after Holm-Bonferroni corrections are indicated in bold font (P < 0.05/N_s_)

## Discussion

The present longitudinal study is the first to show that recovery of smoothness reflected by the *spectral arc length* (SPARC) is highly associated with recovery of FM-UE within moderately to mildly affected patients early after stroke. Both measures show a non-linear time course, with the greatest change taking place within the first 5 weeks post stroke, whereafter their recovery gradually levels off. The significant longitudinal association between SPARC and FM-UE within subjects and their similar time window of recovery of 5 weeks post stroke suggest that their recovery may be driven by a common underlying process responsible for spontaneous neurological recovery early post stroke.

Our findings show that the recovery of smoothness during a multi-joint reaching movement, as quantified by SPARC, follows a similar time course as recovery from motor impairment, as reflected by FM-UE scores, within the first 6 months post stroke. Therefore, this objective kinematic metric reflecting smoothness may be an alternative for clinical measures to reflect motor impairment.

Besides this likely similar time course, we showed a longitudinal within-subject association between SPARC and FM-UE. The yielded within-subject regression coefficient estimate reflects the degree of increase of one variable when the other variable increases with 1.0 within a subject [38]. Our findings show that observed time-dependent changes of smoothness and recovery of FM-UE scores are associated with each other within subjects. These findings suggest that both measures may be driven by the same underlying processes of spontaneous neurological recovery. Despite the likely similar time courses of SPARC and FM-UE, and longitudinal within-subject association, the underlying neurophysiological cause of diminished smoothness after stroke remains unclear and requires further investigation.

The lower movement smoothness observed in the investigated group of mildly to moderately affected patients at 6 months post stroke, compared to reference values of age-matched healthy individuals, suggests that residual movement smoothness deficits remain present in most patients who suffered a stroke. The Stroke Recovery and Rehabilitation Roundtable task force (SRRR) [40] suggested that kinematics quantifying QoM may have added informative value to identify minor deficits in those who show full motor recovery based on clinical assessments. In our sample, the number of patients that show full recovery based on FM-UE scores was too small to perform a sufficient-powered analysis to determine whether smoothness quantified as SPARC is a more responsive biomarker to identify remaining motor impairments when compared to FM-UE. SPARC as a marker for full sensorimotor recovery requires further investigation.

It is important to note that during recovery early after stroke, not all kinematic metrics improve and follow a non-linear time course. For example, endpoint accuracy of the hand during reaching, shows a poor longitudinal association with FM-UE [7]. Obviously, a metric that allows multi-joint compensation strategies during reaching prevents to measure ‘true’ neurological recovery post stroke. This finding suggests that understanding how uniquely a metric reflects underlying neurological impairment, is an important feature for designing stroke recovery and rehabilitation trials investigating quality of movement early post stroke, as recently emphasized by the SRRR [40].

### Limitations

Only patients who were moderately to mildly affected due to a stroke were included in the present study since participants had to be able to perform the reach-to-grasp task within 3 weeks post stroke. Despite this attrition bias in patient selection, the current longitudinal study strongly suggests that recovery of FM-UE closely parallels recovery of smoothness and levels off after 5 weeks post stroke. Such a restricted time window has been shown to be typical of this subpopulation [5]. The generalisability of our findings is restricted to smoothness of reach-and-grasp tasks performed using a block of 5×5×5cm. This object could be picked-up by most patients, and thereby resulted in the most complete dataset. When using larger objects, one should consider the weight of the object since strength is a confounder for motor control during reaching after stroke [30]. A reach-to-point task, not requiring the ability to grasp, would allow for smoothness to be measured in more severely affected patients, reducing the attrition bias. In addition, currently, consensus on how to determine the exact end of a reaching movement is lacking. Our method is in line with the approach as described by Alt Murphy et al. [37], and Michaelsen and Levin [36]. Secondly, the present analyses used FM-UE total scores, which also assess the functioning of the fingers, while pathological synergisms are mainly present in the more proximal part of the upper extremity (i.e., wrist, elbow, shoulder). However, we found similar associations when FM-UE hand scores were ignored (Additional file 1: Section C). Finally, earlier studies suggested that recovery of smoothness deficits reflects neurological recovery [11, 16], which is in line with the findings of the present study. However, in contrast to the performance assays recommended by the SRRR [40], smoothness during multi-joint movements may be influenced at different degrees of motor control. In these cases, the underlying neurophysiological cause remains unclear and its association with compensation strategies cannot be ruled out. Therefore, we recommend to also measure smoothness during single-joint experiments, preventing compensation strategies.

### Future directions

Although smoothness is seen as an important measure of movement quality, recovery from smoothness deficits after stroke is poorly understood. The present findings do not rule out any hypothesized cause of smoothness deficits early post stroke. Determining which neurophysiological deficit after stroke is the main cause of decreased smoothness requires further investigation. One might think of combining repeated measurements of kinematics to measure smoothness, with EMG to determine muscle activity patterns, and non-invasive neuroimaging techniques such as MRI, fMRI or DTI.

Although FM-UE is considered to be a clinical measure for assessment of muscle synergies during recovery post stroke, it is important to note that FM-UE is not purely measuring muscle synergies. The systematic coupling by co-activation of muscles across multiple joints are influenced by strength [30]. Thereby, strength is a confounding factor for the true coupling between different joints during reaching [41]. How the increased muscle synergies during reaching after stroke are longitudinally associated with SPARC within subjects remains to be investigated. For example, Ellis and colleagues showed that increased shoulder-elbow coupling is associated with reduced work area during a 2D drawing task, while the work area improves by arm-weight support [30]. In a similar way, Bartolo and colleagues showed that arm-weight support in robotics results in a significantly reduced amount of jerk [42]. Therefore, we suggest to repeat our measurements using weight support. In addition, a more advanced method than the FM-UE is required to quantify muscle synergies, which prevents against the confounding influence of strength and does not suffer from ceiling effects [30, 41]. This may enable to investigate the longitudinal within-subject association between muscle synergies and smoothness after stroke.

Smoothness is used as reflection of quality of movement and the degree of motor control in many studies. In line with the findings in the present study, recovery of smoothness deficits after stroke has been suggested to be associated with neurological recovery. Therefore, SPARC may serve as outcome measure in studies which investigate the effect of interventions such as upper limb robotics or brain stimulation. In the present study, statements about similarity in time course of recovery are based on visual inspection and the determined time window of recovery. However, further mathematical underpinning is necessary to determine whether the time course of SPARC and FM-UE post stroke are truly similar (e.g., by performing an exponential fit or principal component analysis [43]).

We recommend that future kinematic studies investigating smoothness during multi-joint reaching movements use SPARC. We showed previously, by performing simulation analyses, that SPARC meets the requirements of internal validity to reflect smoothness during reaching tasks [24]. In the present study, we examined the external validity based on longitudinal data of stroke patients who perform a reach-to-grasp task. Furthermore, to determine whether smoothness can serve as a performance assay, the improvement of smoothness should be related to true neurological repair in absence of compensation strategies. For this latter aim, the motor paradigm should focus on performing a single-joint task.

Healthy individuals, especially the elderly, may also show deviations from the optimal reaching trajectory, resulting in a certain decrease of smoothness [44, 45]. Studies should include reference data from age-matched healthy subjects, in order to determine whether smoothness values are significantly different from what could be expected in healthy state. Obviously, the tasks performed should be similar in order to be able to compare smoothness values. Hence, no general task-independent cut-off to distinguish between normal and abnormal smoothness could be provided in the present study.

Finally, repeated measurements within subjects, which are required for stroke recovery studies, are highly demanding for participants. To limit the burden for patients by reducing preparation time and enabling measurements to take place at their place of residence, ambulant measurement systems for measuring smoothness should be simplified. Recently, it was shown that an ambulant system based on inertial measurement units was not capable of measuring SPARC for translational movements due to issues of drift commonly seen in these systems [46]. Further development of simple ambulant measurement systems is needed to enable valid and reliable measurements using wearables.

## Conclusions

The present findings show that the recovery of smoothness during a multi-joint reaching task reflected by SPARC and the recovery from motor impairment reflected by FM-UE are longitudinally associated and highly likely to follow a comparable time course. This finding suggests that the reduction of smoothness deficits quantified by SPARC is a proper objective reflection of recovery from motor impairment, as reflected by FM-UE, and may be driven by a common underlying process of spontaneous neurological recovery within the first 5 weeks post stroke in patients who are moderately to mildly affected due to a stroke.

## Supplementary Information

**Additional file 1.** A. Time course of smoothness and upper extremity motor impairment scores, an overview of all time points against each other. B. Overview of data used for statistical analyses: kinematics, clinical scores and patient characteristics. C. Sub-analysis of FM-UE without hand scores: recovery over time and longitudinal association with SPARC.

## Data Availability

The datasets supporting the conclusions of this article are included within the article and its additional file.

## Abbreviations

FM-UE: Fugl-Meyer motor assessment of the upper extremity
SPARC: Spectral arc length
QoM: Quality of movement
