# Supplementary material for: Smoothness metric during reach-to-grasp after stroke: part 2. longitudinal association with motor impairment
Source: J Neuroeng Rehabil. 2021 Sep 24;18:144. doi: 10.1186/s12984-021-00937-w (PMC8461930; doi:10.1186/s12984-021-00937-w)
Supplement: Supplementary file 1 — Additional file 1. A. Time course of smoothness and upper extremity motor impairment scores, an overview of all time points against each other. B. Overview of data used for statistical analyses: kinematics, clinical scores and patient characteristics. C. Sub-analysis of FM-UE without hand scores: recovery over time and longitudinal association with SPARC. [file 12984_2021_937_MOESM1_ESM.pdf]

Supplementary materials

A. Time course of smoothness and upper extremity motor impairment scores, an overview of all time points against each other.

Time course of SPARC post stroke

SPARC	*Week 1*			*Week 2*			*Week 3*			*Week 4*		
	B	*95%-CI*	*P*	*B*	*95%-CI*	*P*	*B*	*95%-CI*	*P*	*B*	*95%-CI*	*P*
Week 1	-	-	-									
Week 2	0.08	[0.01 – 0.14]	**0.017**	-	-	-						
Week 3	0.17	[0.11 – 0.23]	**<0.001**	0.09	[0.03 – 0.14]	**0.002**	-	-	-			
Week 4	0.20	[0.13 – 0.26]	**<0.001**	0.12	[0.06 – 0.18]	**<0.001**	0.03	[-0.02 – 0.08]	0.245	-	-	-
Week 5	0.21	[0.15 – 0.27]	**<0.001**	0.13	[0.08 – 0.19]	**<0.001**	0.04	[-0.01 – 0.09]	0.083	0.01	[-0.04 – 0.07]	0.624
Week 8	0.23	[0.17 – 0.29]	**<0.001**	0.15	[0.09 – 0.21]	**<0.001**	0.06	[0.01 – 0.12]	**0.019**	0.03	[-0.02 – 0.09]	0.254
Week 12	0.24	[0.18 – 0.31]	**<0.001**	0.16	[0.11 – 0.22]	**<0.001**	0.08	[0.03 – 0.13]	**0.003**	0.04	[-0.01 – 0.10]	0.094
Week 26	0.26	[0.20 – 0.32]	**<0.001**	0.18	[0.13 – 0.23]	**<0.001**	0.09	[0.04 – 0.14]	**<0.001**	0.06	[0.01 – 0.11]	**0.025**
	Week 5			*Week 8*			*Week 12*			*Week 26*		
	B	*95%-CI*	*P*	*B*	*95%-CI*	*P*	*B*	*95%-CI*	*P*	*B*	*95%-CI*	*P*
Week 1												
Week 2												
Week 3												
Week 4												
Week 5	-	-	-									
Week 8	0.02	[-0.03 – 0.07]	0.478	-	-	-						
Week 12	0.03	[-0.02 – 0.08]	0.210	0.01	[-0.04 – 0.07]	0.623	-	-	-			
Week 26	0.05	[-0.00 – 0.10]	0.062	0.03	[-0.02 – 0.08]	0.290	0.01	[-0.04 – 0.06]	0.558	-	-	-

Abbreviations: B. regression coefficient; 95%-CI. 95% confidence interval; P. probability value; SPARC. spectral arc length adapted.

Time course of FM-UE post stroke

FM-UE	*Week 1*			*Week 2*			*Week 3*			*Week 4*		
	B	*95%-CI*	*P*	*B*	*95%-CI*	*P*	*B*	*95%-CI*	*P*	*B*	*95%-CI*	*P*
Week 1	-	-	-									
Week 2	11.59	[8.81 – 14.37]	**<0.001**	-	-	-						
Week 3	16.01	[13.02 – 19.01]	**<0.001**	4.42	[1.38 – 7.46]	**0.005**	-	-	-			
Week 4	18.22	[15.29 – 21.16]	**<0.001**	6.63	[3.65 – 9.62]	**<0.001**	2.21	[-0.97 – 5.40]	0.173	-	-	-
Week 5	18.86	[16.12 – 21.59]	**<0.001**	7.27	[4.47 – 10.07]	**<0.001**	2.85	[-0.16 – 5.85]	0.063	0.64	[-2.31 – 3.58]	0.674
Week 8	20.53	[17.68 – 23.38]	**<0.001**	8.94	[6.03 – 11.85]	**<0.001**	4.52	[1.41 – 7.63]	**0.005**	2.31	[-0.75 – 5.36]	0.138
Week 12	20.82	[18.04 – 23.60]	**<0.001**	9.23	[6.39 – 12.07]	**<0.001**	4.81	[1.77 – 7.85]	**0.002**	2.60	[-0.38 – 5.58]	0.087
Week 26	21.48	[18.76 – 24.19]	**<0.001**	9.89	[7.11 – 12.67]	**<0.001**	5.46	[2.47 – 8.46]	**<0.001**	3.25	[0.32 – 6.18]	**0.030**
	Week 5			*Week 8*			*Week 12*			*Week 26*		
	B	*95%-CI*	*P*	*B*	*95%-CI*	*P*	*B*	*95%-CI*	*P*	*B*	*95%-CI*	*P*
Week 1												
Week 2												
Week 3												
Week 4												
Week 5	-	-	-									
Week 8	1.67	[-1.19 – 4.53]	0.251	-	-	-						
Week 12	1.96	[-0.83 – 4.75]	0.168	0.29	[-2.61 – 3.19]	0.844	-	-	-			
Week 26	2.62	[-0.12 – 5.35]	0.061	0.94	[-1.91 – 3.79]	0.515	0.66	[-2.12 – 3.43]	0.643	-	-	-

Abbreviations: B, regression coefficient; 95%-CI. 95% confidence interval; P, probability value; FM-UE, Fugl-Meyer motor assessment of the upper extremity.

B. Overview of data used for statistical analyses: kinematics, clinical scores and patient characteristics.

Kinematics - smoothness

SPARC of stroke patients at each measurement moment

ID	Week 1	Week 2	Week 3	Week 4	Week 5	Week 8	Week 12	Week 26
**1**	-1.518		-1.414		-1.466			-1.415
**2**	-1.540	-1.477	-1.512		-1.471	-1.454	-1.482	-1.463
**3**			-1.482		-1.508	-1.460	-1.506	-1.500
**4**		-2.038	-1.789		-2.097	-1.784	-1.720	-1.642
**5**		-1.517	-1.454	-1.448	-1.479	-1.449	-1.427	-1.451
**6**		-1.543	-1.517	-1.525	-1.488	-1.448	-1.458	-1.566
**7**	-2.008	-1.562	-1.566	-1.481	-1.468	-1.450	-1.458	-1.439
**8**	-1.562	-1.427	-1.426	-1.410	-1.435		-1.441	-1.425
**9**	-1.716	-1.716	-1.654	-1.548	-1.677	-1.646	-1.567	-1.572
**10**	-1.516	-1.547	-1.451		-1.462	-1.532	-1.476	-1.443
**11**	-1.452		-1.462	-1.442	-1.421	-1.422	-1.415	-1.423
**12**	-2.413		-1.475	-1.479	-1.498	-1.446	-1.443	-1.429
**13**			-1.754	-1.750	-1.744	-1.565	-1.479	-1.441
**14**	-1.632	-1.499	-1.436	-1.479	-1.508	-1.479	-1.497	-1.463
**15**	-1.866	-1.481	-1.578	-1.453	-1.514	-1.421	-1.444	-1.443
**16**		-1.475	-1.423	-1.425	-1.409	-1.431	-1.420	-1.478
**17**			-1.863	-1.546	-1.444	-1.542	-1.446	-1.433
**18**	-1.649	-1.602	-1.557	-1.551	-1.592		-1.554	-1.547
**19**			-1.523	-1.461	-1.467	-1.519	-1.493	-1.441
**20**			-1.700	-2.160	-1.587	-1.445	-1.439	-1.429
**21**	-1.510	-1.487	-1.508	-1.484	-1.422	-1.497	-1.486	-1.436
**22**			-1.859	-1.526	-1.516	-1.611	-1.492	-1.497
**23**	-2.105	-1.809	-1.661	-1.483	-1.559		-1.447	-1.548
**24**		-1.939	-1.540	-1.545	-1.477	-1.487	-1.494	-1.522
**25**	-1.957	-1.612	-1.600	-1.631	-1.565	-1.577	-1.644	-1.541
**26**	-1.737	-1.520	-1.544	-1.516	-1.531	-1.525	-1.523	-1.441
**27**		-2.206	-1.522	-1.484	-1.457	-1.433	-1.435	-1.437
**28**	-1.778	-1.648	-1.601	-1.548	-1.529	-1.460	-1.545	-1.415
**29**		-1.657	-1.647	-1.550	-1.519	-1.539	-1.482	-1.543
**30**	-1.543	-1.482			-1.410			-1.417
**31**	-1.640	-1.579	-1.601	-1.540	-1.514	-1.533	-1.588	-1.485
**32**		-1.798	-1.581	-1.563	-1.566	-1.487	-1.525	-1.470
**33**	-1.641	-1.694	-1.631	-1.803	-1.661	-1.625	-1.673	-1.640
**34**		-1.930	-1.707	-1.456	-1.567	-1.503	-1.460	-1.497
**35**			-1.591	-1.584	-1.483	-1.531	-1.527	-1.486
**36**	-1.593	-1.544	-1.522	-1.449	-1.480	-1.512	-1.442	-1.414
**37**		-1.479	-1.593	-1.570	-1.575		-1.542	-1.500
**38**		-1.677		-1.569	-1.583	-1.599	-1.549	-1.525
**39**		-1.534	-1.491		-1.475	-1.502	-1.507	-1.477
**40**		-2.231	-1.496	-1.451	-1.432		-1.425	-1.452

SPARC of healthy age and gender matched individuals

ID	Mean	Std
**H1**	-1.430	0.012
**H2**	-1.412	0.005
**H3**	-1.462	0.062
**H4**	-1.446	0.045
**H5**	-1.414	0.008
**H6**	-1.420	0.010
**H7**	-1.424	0.015
**H8**	-1.409	0.006
**H9**	-1.434	0.011
**H10**	-1.461	0.078
**H11**	-1.480	0.055
**H12**	-1.437	0.047

Abbreviations: SPARC, spectral arc length adapted; ID, subject number; std, standard deviation.

Clinical scores

FM-UE of stroke patients at each measurement moment

ID	Week 1	Week 2	Week 3	Week 4	Week 5	Week 8	Week 12	Week 26
**1**	60	62		65	66			66
**2**	46	48		48	46	47	50	54
**3**	47	51		56	60	62	60	56
**4**	34	39	41		44	49	56	57
**5**	55			63	64	63	64	64
**6**	9	50	64	63	65	66	66	66
**7**	46	55	59	62	62	59	60	63
**8**	53	62	65	66	66		66	66
**9**	52	61	61	61	62		63	66
**10**	60	65	66	66	66	66	66	66
**11**	60	64		65	66	66	66	66
**12**	39	47	58	63	62	62	62	59
**13**	19	35	43	45	46	47	51	63
**14**	60	62	63	63	63	64	65	64
**15**	58	60	61	62	63	64	64	64
**16**	58	65	66		66	66	66	66
**17**	35		50	62	62	65	66	65
**18**	30	52	53	53	49		44	49
**19**	63	65	65	66	66	66	66	66
**20**	23	45	49	52	62	65	66	66
**21**	62	63	63	64	65	64	64	65
**22**	5	26		53	56	58	59	59
**23**	35	49	53	58	59	63	63	59
**24**	13	41	45	49	51	62	63	65
**25**	18	49	51	54	54	56	55	56
**26**	38	52	61	62	64		64	65
**27**	35				63	65	65	65
**28**	43	48	55	54	57	59	59	61
**29**	19	28		51	55	58	58	58
**30**	48	51						56
**31**	44	49	56	56	56	56	63	64
**32**	31	41	46		46	52	48	49
**33**	38	42	42		41	35	39	47
**34**	44	49	50		52	57	60	63
**35**	29	46	55		57	58		60
**36**	46	60	60	61	63	63	62	61
**37**	27	51	60	64	64	66	66	63
**38**	48	60		62	63	63	64	63
**39**	56	58			62	65	65	65
**40**	11	47	52	54	58	62	58	60

Abbreviations: FM-UE, Fugl-Meyer motor assessment of the upper extremity [0 – 66]; ID, patient number.

Patient characteristics

ID	Sex (male/female)	Age	Affected body side (left/right)	Hand dominance (left/right/forced right)	Bamford classification (LACI/PACI/TACI)
**1**	female	57	left	right	LACI
**2**	male	67	right	right	PACI
**3**	female	45	left	right	TACI
**4**	male	54	left	right	LACI
**5**	male	32	left	right	LACI
**6**	male	71	right	right	PACI
**7**	male	62	left	left	LACI
**8**	female	79	right	right	LACI
**9**	female	74	left	right	LACI
**10**	male	38	left	right	LACI
**11**	female	64	right	forced right	LACI
**12**	female	75	right	right	LACI
**13**	male	51	right	right	LACI
**14**	female	74	left	right	LACI
**15**	female	63	left	right	LACI
**16**	female	71	left	right	LACI
**17**	male	39	right	right	PACI
**18**	male	64	left	right	PACI
**19**	male	56	left	right	PACI
**20**	male	41	right	right	LACI
**21**	male	36	left	right	LACI
**22**	male	56	left	right	LACI
**23**	male	50	left	right	PACI
**24**	male	63	left	right	LACI
**25**	female	57	left	right	LACI
**26**	female	52	right	right	LACI
**27**	female	40	left	right	LACI
**28**	male	69	right	right	LACI
**29**	male	44	right	left	PACI
**30**	female	70	left	right	LACI
**31**	female	69	right	right	PACI
**32**	female	46	left	right	TACI
**33**	female	68	left	right	LACI
**34**	male	73	right	right	PACI
**35**	male	60	left	right	LACI
**36**	male	66	left	right	LACI
**37**	female	51	left	right	LACI
**38**	male	65	left	right	LACI
**39**	male	59	right	right	LACI
**40**	female	72	right	right	LACI

Abbreviations: ID, patient number; LACI, lacunar anterior circular infarct; PACI, partial anterior circular infarct; TACI, total anterior circular infarct.

C. Fugl-Meyer assessment of the upper extremity – without hand scores

Sub-analysis of time course of Fugl-Meyer while hand scores were ignored, and its longitudinal association with SPARC. This refers to a sub-analysis mentioned in the discussion section. The longitudinal association with SPARC is similar.

Time course of FM-UE without hand scores.

FM-UE without hand	*Week 26*		
	B	*95%-CI*	*P*
Intercept	47.8	[45.4 – 50.2]	**<0.001**
Week 1	-16.2	[-18.4 – -14.0]	**<0.001**
Week 2	-7.6	[-9.8 – -5.4]	**<0.001**
Week 3	-4.4	[-6.8 – -2.0]	**<0.001**
Week 4	-2.7	[-5.0 – -0.4]	**0.022**
Week 5	-2.4	[-4.6 – -0.2]	**0.032**
Week 8	-0.7	[-2.9 – 1.6]	0.553
Week 12	-0.6	[-2.8 – 1.6]	0.568
Week 26	-	-	-

Maximum scores of FM-UE without hand is 52.

Longitudinal associations between FM-UE without hand scores and SPARC.

Regular mixed model analysis	*Longitudinal association with FM-UE without hand scores*		
	B	*95%-CI*	*P*
SPARC	23.0	[19.2 – 26.8]	**<0.001**

Hybrid mixed model analysis	*Longitudinal association with FM-UE without hand scores*		
	B	*95%-CI*	*P*
Within subjects effect	22.4	[18.6 – 26.3]	**<0.001**
Between subjects effect	40.0	[19.2 – 60.9]	**<0.001**
